# Translation initiation and its relevance in colorectal cancer

**DOI:** 10.1111/febs.15690

**Published:** 2021-01-24

**Authors:** Emma Minnee, William James Faller

**Affiliations:** ^1^ Division of Oncogenomics Netherlands Cancer Institute Amsterdam The Netherlands

**Keywords:** colorectal cancer, protein synthesis, RNA translation, translation initiation, translational control

## Abstract

Protein synthesis is one of the most essential processes in every kingdom of life, and its dysregulation is a known driving force in cancer development. Multiple signaling pathways converge on the translation initiation machinery, and this plays a crucial role in regulating differential gene expression. In colorectal cancer, dysregulation of initiation results in translational reprogramming, which promotes the selective translation of mRNAs required for many oncogenic processes. The majority of upstream mutations found in colorectal cancer, including alterations in the WNT, MAPK, and PI3K\AKT pathways, have been demonstrated to play a significant role in translational reprogramming. Many translation initiation factors are also known to be dysregulated, resulting in translational reprogramming during tumor initiation and/or maintenance. In this review, we outline the role of translational reprogramming that occurs during colorectal cancer development and progression and highlight some of the most critical factors affecting the etiology of this disease.

## Graphical abstract

Colorectal cancer is defined by sequential mutations in a number of signaling pathways, including WNT–, MAPK–, TGFβ–, and p53–pathways. The most common mutations are in the *APC* and *KRAS* genes, causing activation of WNT– and MAPK–signaling respectively. Both of these pathways regulate mRNA translation via multiple downstream effectors, and altered protein synthesis has been shown to be vital for the development and progression of this disease.

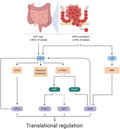

**Abbreviations**
4E‐BP1: eIF4E‐binding protein
5'UTR: 5’‐untranslated region
APC: adenomatous polyposis coli
ATF4: activating transcription factor 4
BCL2: B‐cell lymphoma 2
CCND1: cyclin D1
CITE: cap‐independent translation enhancer
CMS: consensus molecular subtype
CRC: colorectal cancer
eIF: eukaryotic translation initiation factor
ER: endoplasmic reticulum
GCN2: general control nonderepressible 2
GEF: guanine nucleotide exchange factor
GRP78: 78‐kDa glucose‐regulated protein
HRI: heme‐regulated eukaryotic initiation factor EIF‐2alpha kinase
IRES: internal ribosome entry site
ISR: integrated stress response
ITAF: IRES trans‐acting factor
KRAS: Kirsten RAS
MAPKAPK: MAPK‐activated protein kinase
MNK: MAPK‐interacting kinase
mRNA: messenger RNA
mTORC1: mechanistic target of rapamycin complex 1
ODC1: ornithine decarboxylase
PABP: poly(A)‐binding protein
PDCD4: programmed cell death 4
PERK: protein kinase R‐like endoplasmic reticulum kinase
PI3K/AKT: phosphoinositide 3‐kinase/protein kinase B
PIC: preinitiation complex
PKR: protein kinase R
RAS/MAPK: RAS/mitogen‐activated protein kinase
RP: ribosomal protein
rRNA: ribosomal RNA
RSK: p90 ribosomal S6 kinase
S6K1: 70‐kDa ribosomal S6 kinase 1
SPARC: secreted protein acidic and cysteine‐rich
TC: ternary complex
uORF: upstream open reading frame
VEGFA: vascular endothelial growth factor A
WNT: Wingless and Int‐1

## Introduction

Protein synthesis, via the translation of messenger RNA (mRNA), is the most energy‐consuming processes in the human body [1, 2]. It is highly regulated, and flexibility in this regulation is known to be necessary for many complex processes, such as embryonic development, cellular differentiation, and the response to stress [3, 4]. Many studies have shown that steady‐state mRNA levels are not sufficient to predict the proteome, and although there is some controversy about this, the prevailing view is that translational control provides a crucial layer of regulation in defining the ultimate cellular abundance of proteins [5, 6, 7].

The process of RNA translation can be divided into distinct phases, known as translational initiation, elongation, termination, and ribosome recycling. Initiation is considered to be the rate‐limiting phase and requires numerous individual specialized ribosomal components and eukaryotic translation initiation factors (eIFs), as described below [8].

Previous studies have revealed that the dysregulation of the translational process is a crucial driving force of tumorigenesis and progression in various types of cancer [9, 10, 11]. In order to support various oncogenic phenotypes, transformed cells alter their protein synthesis through the selective translation of specific mRNAs [11]. Indeed, almost all signaling pathways known to be important for tumorigenesis converge on translational regulation, and it may provide a promising target for therapeutic intervention [12, 13]. In colorectal cancer (CRC), alterations in WNT, c‐Myc, and KRAS signaling have all been shown to result in the dysregulation of translational, which is essential for intestinal tumorigenesis [14, 15, 16].

CRC is the third most commonly diagnosed cancer in the world. With approximately 881 000 estimated deaths each year, it is also known to be the second leading cause of cancer‐related deaths worldwide [17]. As CRC presents with a rather complex evolutionary process, combined with a heterogeneous phenotype, it is a highly difficult disease to target therapeutically [18]. In recent years, this complexity has been somewhat classified, with the stratification of CRCs into biologically defined consensus molecular subtypes (CMSs) [18, 19, 20, 21]. These studies identified four subtypes, based on a large number of clinical and biological characteristics. The relevance of this classification to dysregulated protein synthesis is largely unknown; however, two subtypes (CMS 2 and CMS 3) are primarily defined by alterations in WNT/c‐Myc signaling and KRAS mutations, respectively, both of which are directly implicated in translational control. Like most cancers, mRNA translation is commonly dysregulated in CRC [22, 23]. Indeed, the second most common alteration found in this disease is the loss of a specific rRNA modification (the m1acp3Ψ on the 18S rRNA); however, the relevance of this is currently unknown [24].

Despite decades of discovery and investigation, to date no approved treatments targeting the translation machinery are currently utilized for therapy in CRC patients [9, 25]. Since evidence points to the importance of translational control in tumor development and progression in CRC, better insight into this topic might help identify potential therapeutic targets. In this review, we will provide an overview of translational control in CRC and how this explicitly affects the etiology of this disease. Although there is still much to be understood in this subject, we will summarize and discuss the most recent findings, clarifying the overall concept of translational control in CRC. In particular, we discuss the mechanisms that regulate the translation machinery, highlighting the importance of translation initiation in translational control. Additionally, we will examine the dysregulation of translational control, focusing on the alterations found in the upstream regulating pathways, as well as in the translation machinery itself, and their role regarding tumorigenesis in CRC.

### mRNA translation initiation

**Fig. 1:**
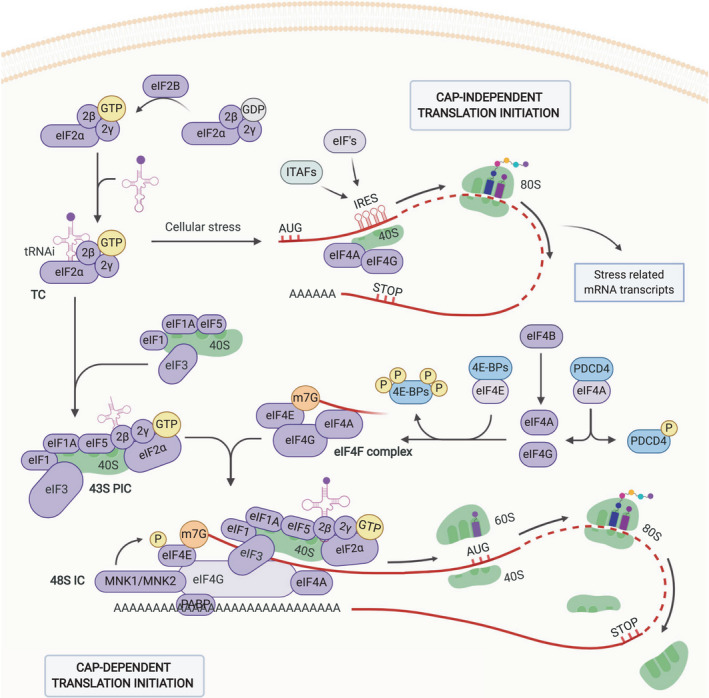
Eukaryotic translation initiation. Schematic representation of eukaryotic translation initiation, including cap‐dependent and cap‐independent translation initiation. The primary mode of initiation is cap‐dependent translation; however, this mechanism can be suppressed in response to a variety of pathophysiological stressors. Cap‐dependent initiation involves the formation of the 43S PIC, followed by the recruitment of the mRNA template facilitated by the eIF4F complex. Once activated, the 43S PIC scans along the 5’UTR of the transcript until the initiator codon (AUG) is recognized, resulting in the release of various eIFs and the formation of the translation competent 80S ribosome. In response to specific stressors, the cell is able to adapt its gene expression pattern through reprogramming of the protein synthesis, using the cap‐independent translation initiation such as IRES‐mediated initiation. These structured mRNA sequences downstream of the 5’UTR m^7^G‐cap can regulate cap‐independent translation initiation via various mechanisms using both the canonical eIFs and IRES *trans*‐acting factors. The exact mechanisms and detailed explanations of the translation initiation machinery are provided in the text. Created with BioRender.com.

Together, the synthesis, accumulation, and translation of mRNA transcripts combine to regulate the cellular abundance of diverse proteins in cells, tissues, and organisms [3, 6, 26]. Translational control is vital for regulating differential gene expression, especially in response to local and systemic changes in the cellular environment, such as stress, aging, and disease [27]. In eukaryotic cells, protein synthesis can be initiated through several mechanisms, including cap‐dependent and cap‐independent translation initiation (Fig. 1) [8, 28]. The primary mode of translation initiation is cap‐dependent translation; however, this mechanism of translation is partially suppressed in response to a variety of pathophysiological stressors [29, 30]. Under such circumstances, the cell can rapidly adjust its protein synthesis, through both cap‐dependent reprogramming and the use of cap‐independent translation initiation, which promotes the upregulation of genes important for the cellular stress response [31]. It is known that the dysregulation of this process leads to alterations in the homeostasis and maintenance of the cell. Hence, the strict regulation of this cellular process is crucial [9, 10].

### Cap‐dependent translation initiation

The predominant form of eukaryotic translation initiation requires the recognition of the m^7^G‐cap of the 5’‐untranslated region (5’UTR) of an mRNA transcript, followed by ribosomal scanning [8]. Various sequence elements coordinate and facilitate the guidance of at least 12 eIFs and their associated ribosomes, with the goal of forming the elongation component 80S ribosome and beginning protein synthesis [28].

Cap‐dependent translation is initiated by the formation of a multifactorial 43S preinitiation complex (PIC) via the association of the 40S ribosomal subunit with initiation factors eIF1, eIF1A, eIF3, and eIF5 and the ternary complex (TC), which itself is composed of a GTP‐bound eIF2 and Met‐tRNA_i_. This pre‐assembled 43S PIC is then recruited to the m^7^G‐cap, facilitated by eIF4F, forming the 48S preinitiation complex. eIF4F itself is a complex consisting of a mRNA 5’‐cap‐binding subunit eIF4E, the scaffold protein eIF4G, and the RNA helicase eIF4A [8, 31, 32]. As part of the 48S complex, eIF4G also facilitates the interaction of the mRNA 3’‐poly(A) tail with the poly(A)‐binding protein (PABP), which results in the circularization of the transcript in order to stabilize the mRNA and augment translation [33, 34]. Once the 48S complex binds near the preactivated m^7^G‐cap of the mRNA transcript, it scans along the 5’UTR toward the 3’‐end through codon–anticodon base pairing in an ATP‐dependent fashion, until the start codon (AUG) is encountered [35, 36, 37]. The recognition of the AUG codon triggers a global conformational change in the 40S ribosomal subunit, from a scanning competent to a scanning‐arrested state, which allows codon–anticodon base pairing with Met‐tRNA_i_. This assembly initiates the release of the phosphate from the GTP, hydrolyzed by eIF2, leading to a reduced affinity of eIF2 for the tRNA and its subsequent release [38, 39]. Immediately after the release of eIF2, eIF5B‐GTP binds to the same domain on the 48S complex, creating an increased 60S/40S‐interacting surface, which allows the recruitment of the large 60S ribosomal subunit [39, 40, 41, 42, 43]. The correct positioning of the 60S large ribosomal subunit on the initiation complex forms the elongation component 80S ribosome, which stimulates the eIF5B‐GTP hydrolysis resulting in the dissociation of eIF5B‐GTP and any other remaining initiation factors [40, 44]. This dissociation of eIF5B demarcates the end of the initiation phase of the translation, which then proceeds into translation elongation [45].

### Cap‐independent translation initiation

A second form of translation initiation exists and is usually associated with the translation of mRNA transcripts following environmental and cellular stresses [46, 47]. In response to specific stressors, including nutrient deprivation, hypoxia, endoplasmic reticulum (ER) stress, and DNA damage, the cell is able to adapt its gene expression pattern through such reprogramming of protein synthesis [30]. This translational reprogramming relies on the upstream activation of assorted stress‐related kinases and phosphatases (which are specific to the stress driving the reprogramming), leading to the modulation of the phosphorylation states of key regulating eIFs, most important of which is eIF2α [48]. As a result of this phosphorylation, cap‐dependent translation is suppressed, while cap‐independent translation is largely unaffected. This results in the preferential translation of individual stress‐related mRNAs and is a mechanism the cell can use to overcome a general repression of cap‐dependent protein synthesis [8, 49]. This process is highly regulated, and its dysregulation is commonly associated with tumor formation and cancer development.

Multiple physiological stressors can induce an integrated stress response (ISR), primarily via signaling cascades that result in the activation of various eIF2α kinases, and their activation results in the phosphorylation of the eIF2 α‐subunit, eIF2α [48]. This phosphorylation increases the affinity of eIF2‐GDP for eIF2B, a guanine nucleotide exchange factor (GEF), which thereby sequesters the eIF2B GEF activity and availability required for the formation of the 43S PIC during canonical translation initiation [50, 51, 52]. This not only results in an inhibition of canonical cap‐dependent translation initiation, but also counterintuitively stimulates the cap‐dependent translation of a small subset of mRNAs encoding proteins involved in the ISR. This subset of mRNAs contain an upstream open reading frame (uORF), allowing translation initiation of mRNAs encoding stress‐related proteins, including ATF4, triggering the cell’s stress response [53].

A common alternative to cap‐dependent recruitment of the translation machinery is the use of internal ribosome entry site (IRES)‐mediated cap‐independent translation. This mechanism relies on the direct recruitment of the ribosomes to the IRES sequence [54]. IRES elements are diverse but structured mRNA sequences downstream of the 5’UTR m^7^G‐cap that regulate cap‐independent translation initiation via various mechanisms using both the canonical eIFs and IRES *trans*‐acting factors (ITAFs) [55]. The nature of IRES elements is not well understood, and it has been suggested that such structures be renamed ‘cap‐independent translation enhancers’ (CITEs) [56]. However, regardless of this debate [57, 58], evidence suggests that approximately 10% of all mRNAs contain such structures and that these elements are primarily responsible for the recruitment of the ribosomes to the internal region of the mRNA transcript [59]. In cancer, both extrinsic and intrinsic stressors, including hypoxia, nutrient deprivation, and oncogene activation, can promote the IRES‐mediated translation of a selective set of stress‐related mRNA transcripts, among which is the proto‐oncogene c‐Myc, whose activation is a common event in CRC [60].

## Upstream translation initiation regulation in colorectal cancer

Tumor outgrowth depends on a selective protein synthesis that directly determines the enhanced translation of proteins required for oncogenic cellular processes [11]. Accordingly, many studies have shown that this selective protein synthesis is a consequence of the accumulation of genomic alterations in oncogenes or tumor suppressors, and CRC is no exception to this. The majority of CRC cases are initiated by the accumulation of specific mutations within the intestinal tract, eventually leading to the progression of benign polyps/adenomas into malignant carcinomas [61]. The initiating event in the majority of CRC cases is a mutation in the tumor suppressor gene *APC*, promoting the permanent activation of the WNT signaling pathway [62]. APC deficiency drives the transcriptional upregulation of multiple genes, including many associated with protein synthesis and the cellular stress response, accounting for a complicated multistep carcinogenic cascade to ensure tumor cell survival [63]. The dysregulated expression of these WNT target genes is thought to allow tumor outgrowth through oncogenic ‘translational reprogramming’ [64, 65, 66]. The continuous activation of WNT signaling and the reprogramming of translational control are genetic bedrocks for the majority of the CRC cases and are therefore attractive targets for further therapeutic intervention. Accordingly, a better understanding of how WNT‐related alterations might affect the translation initiation process in CRC is of great importance.

**Fig. 2:**
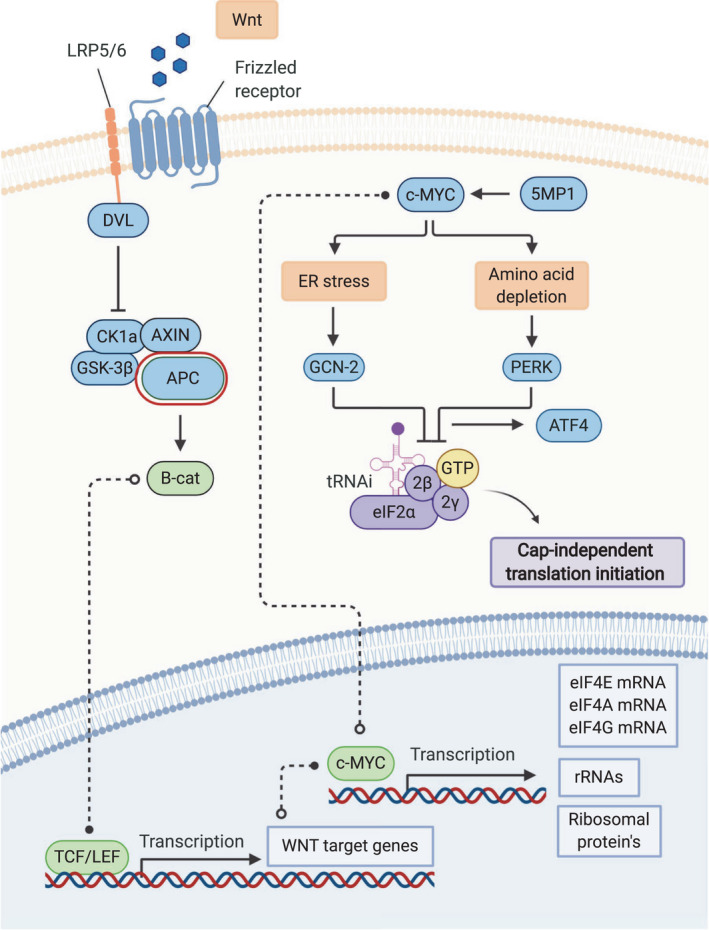
Translational control in CRC. Schematic representation of the translational control in CRC, including the upstream regulatory pathways and the translation initiation machinery. Factors contributing to the translational reprogramming in CRC are mutations and alterations found in the *APC* gene, in genes involved in RAS/MAPK signaling, including *KRAS* and *BRAF*, and in PI3K/AKT signaling, via alterations in PTEN and PI3K (marked in red). In CRC, the translational reprogramming is mainly regulated via the function and activation of the central regulators mTORC1 and c‐Myc. Besides these alterations found in the upstream signaling pathways, the altered expression and activity of numerous eIFs as well as the upregulation of components for ribosomal biogenesis and factors controlling translation in response to stress, are shown to dysregulate the translation initiation machinery in CRC. Positive regulators of the canonical translation initiation machinery are shown in green, while the negative regulators of this process are shown in blue and the individual translation initiation factors are shown in purple. Detailed explanations and descriptions about the translational control and its reprogramming in CRC are provided in the text. Created with BioRender.com.

Among the most commonly described mutations found in CRC are alterations in RAS/MAPK and PI3K/AKT pathway members, driving a subsequent activation of mTOR signaling. These alterations are known to facilitate the reprogramming of the translation machinery [27, 67, 68, 69]. Besides these alterations found in the upstream signaling pathways, the altered expression and activity of numerous eIFs [70] (e.g., eIF4E, eIF4A, eIF2α), as well as the upregulation of ribosomal biogenesis [71] and factors controlling translation in response to stress [72] (e.g., GNC2 and c‐Myc), have all been shown to dysregulate the translation initiation machinery in CRC (Fig. 2). Below, we discuss the most critical mediators affecting the oncogenic translation initiation reprogramming in CRC development and progression.

### c‐Myc as a translation initiation regulator

**Fig. 3:**
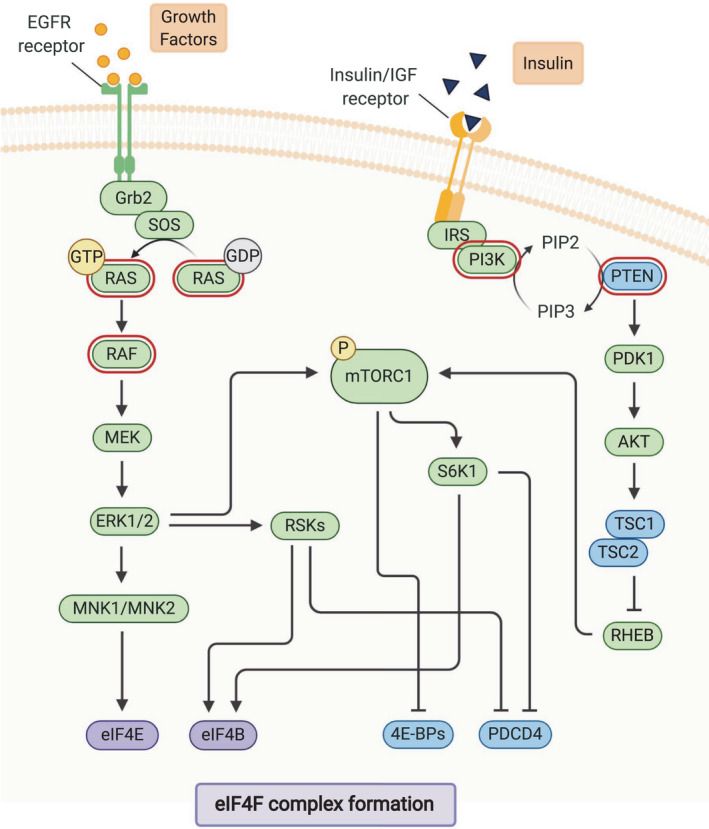
The function of transcription factor c‐Myc in translational control. Schematic representation of the function of c‐Myc during translational reprogramming in CRC. The loss of upstream key regulator APC increases the global translation rates via the overexpression of c‐Myc, through the constitutive accumulation of β‐cat. The constitutive overexpression of c‐Myc stimulates the transcription and activation of major downstream effectors involved in the oncogenic translation initiation machinery. c‐Myc overexpression in CRC can lead to an upregulation of ribosome biogenesis and is implicated in cellular responses to stress via regulating the activity of stress‐related kinases GCN2 and PERK and that of the eIF2α/eIF2B complex, driving cap‐independent translation initiation. Detailed explanations and descriptions about the function of c‐Myc in translational control and its reprogramming in CRC are provided in the text. Created with BioRender.com.

An early event during CRC tumorigenesis is the loss of *APC*, which increases global mRNA translation rates [15]. This results in the transcription of numerous WNT target genes, among which is the proto‐oncogene *c‐Myc* [73], which is thought to be an essential downstream regulator of WNT signaling [74]. It has been shown that c‐Myc is at least partially responsible for the translational reprogramming in CRC, leading to the oncogenic transformation [15]. Accordingly, it has been shown that ribosomal haploinsufficiency is sufficient to block oncogenic capacity of c‐Myc mouse in a model of lymphoma [75]. It is likely that this regulation of protein synthesis, as well as the c‐Myc regulation of stress responses found in CRC cells [72], ensures tumor cell survival (Fig. 3).

As well as regulating translation, c‐Myc itself is known to be regulated by translation. It contains two in‐frame start codons, AUG and CUG, of which the AUG‐initiated c‐Myc isoform shows higher protein stability and activity and is therefore thought to be the more oncogenic isoform compared with its CUG‐initiated counterpart [76, 77, 78]. As a result, the constitutive overexpression of the AUG‐initiated c‐Myc eventually stimulates the transcription of major downstream effectors involved in the oncogenic translation initiation machinery [79]. This translational feedback loop results in a signaling cascade involving GCN2 and the eIF2α/eIF2B complex, driving IRES‐mediated translation initiation [72]. Aside from this alternative translation start site, c‐Myc is also known to both regulate and be regulated by, canonical, eIF4E‐mediated translation. While this has not been shown for CRC, it is known to be crucial for various other malignancies [80, 81, 82], highlighting the centrality of the interplay between c‐Myc signaling and translation in cancer development.

### c‐Myc regulation of ribosome biogenesis

Ribosomes are supramolecular RNA–protein complexes, composed of roughly 80 distinct ribosomal proteins (RPs) and four different ribosomal RNAs (rRNAs), responsible for protein synthesis in every living cell [83, 84]. Compelling evidence indicates that c‐Myc modifies ribosomal biogenesis by controlling the expression of various RPs and rRNAs, thereby regulating translation and protein synthesis in general [85]. It is well known that as a consequence of c‐Myc overexpression, rRNA synthesis is hyperactivated [86]. Recent evidence has also shown that rDNA transcription and subsequent ribosomal biogenesis are elevated specifically within proliferative cells CRCs and that many RPs, including RPL15 and RPS24, are dysregulated, but these have not yet been directly linked to c‐Myc activity [87, 88, 89, 90].

### c‐Myc regulation of translation initiation

In addition to ribosomal biogenesis, c‐Myc can also regulate the transcription of various translation initiation factors [25]. The upregulation of c‐Myc induces the transcription of genes encoding for proteins involved in the translation machinery, such as eIF4E, eIF4A, eIF5A, and eIF4G, via their high‐affinity c‐Myc‐binding sites [20, 91]. However, it is thought that the main role of c‐Myc in the regulation of the translation initiation machinery lies in the coordinated activity of various factors involved in IRES‐dependent translation initiation and eIF2α. In APC‐deficient CRC models, c‐Myc overexpression induces the phosphorylation of eIF2α via the eIF2α kinases GCN2 and PERK. This results in a negative feedback loop, as the expression of c‐Myc is dependent on the activity of eIF2α. The authors show that without this feedback, the cells undergo c‐Myc‐dependent apoptosis, which is prevented by the dampening of c‐Myc translation [72]. It has also been shown that the c‐Myc‐mediated phosphorylation of eIF2α, via GCN2, may also be responsible for the upregulation of the transcription factor ATF4 leading to the enhanced expression of 4E‐BP1, a negative regulator of translation [92].

#### mTORC1 signaling as a translation initiation regulator

**Fig. 4:**
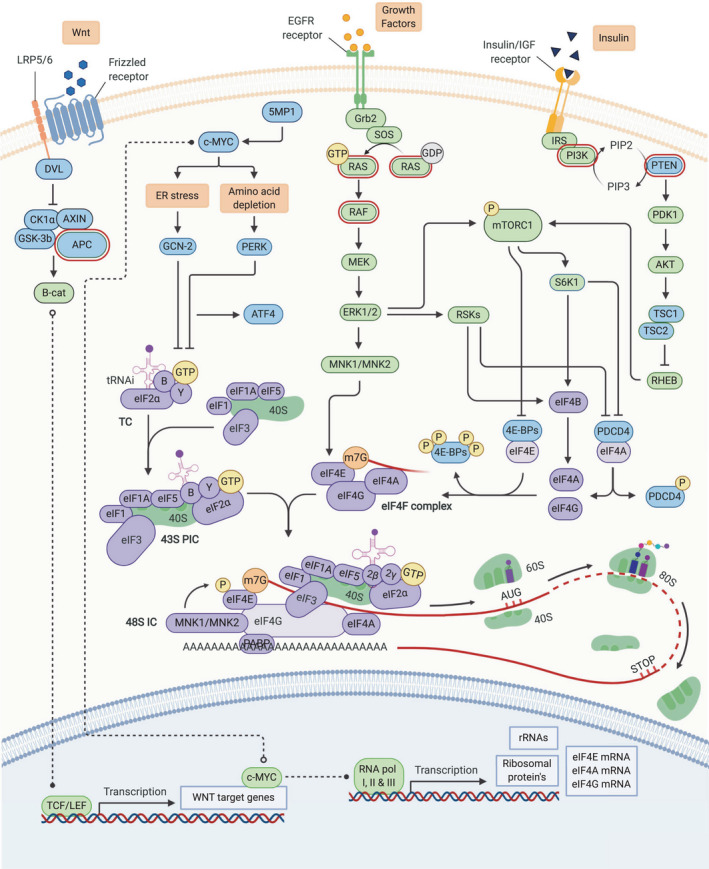
The upstream regulatory pathways controlling translational reprogramming in CRC. Schematic representation of the key upstream regulatory pathways regulating translational control in CRC. mTORC1 lies at the nexus of the major signaling pathways driving translational control, and its activation can be controlled via upstream signaling pathways RAS/MAPK and PI3K/AKT. In CRC, oncogenic signaling via mutations and alterations in these pathways can promote translational reprogramming, predominantly through the altered function of mTORC1 but also via the RAS/MAPK pathway directly. Factors contributing to such hyperactivated mTORC1 signaling are mutations and alterations found in the *APC* gene, in genes involved in RAS/MAPK signaling, including *KRAS* and *BRAF*, and in PI3K/AKT signaling, via alterations in PTEN and PI3K. Detailed explanations and abbreviations about the function of the upstream regulatory pathways in translational control and its reprogramming in CRC are provided in the text. Created with BioRender.com.

mTOR is a conserved serine/threonine (Ser/Thr) protein kinase, which serves as a regulator and coordinator of proliferation and cell growth in response to numerous growth factors, environmental stimuli, cellular energy status, and nutrient/oxygen availability [93]. Over the last few decades, mapping the mTOR signaling landscape has revealed that in addition to c‐Myc, mTORC1 lies at the nexus of all the major signaling pathways driving translational control (Fig. 4) [11]. The activity of mTORC1 is known to be controlled via the upstream signaling pathways RAS/MAPK and PI3K/AKT [27]. In CRC, oncogenic signaling via alterations and mutations in those pathways promotes translational reprogramming, predominantly through the altered function of mTORC1 [22, 94, 95, 96, 97, 98]. As a result, mTORC1 is thought to be hyperactivated in most CRC cases.

The most extensively described mTOR signaling effectors are the eIF4E‐binding proteins (4E‐BPs) [99] and 70‐kDa ribosomal S6 kinase 1 (S6K1) [100, 101]. Since the levels of phosphorylated 4E‐BPs are frequently increased in CRC, and is associated with survival outcomes, this protein is thought to be an important mediator in CRC tumorigenesis [102]. In addition to the 4E‐BPs, phosphorylation of S6K1 has also been identified as a prognostic marker for CRC and has been shown to regulate translation initiation and elongation in models of CRC [15, 103]. Furthermore, S6K1‐mediated activation of eIF4B [104] and degradation of eIF4A inhibitor PDCD4 [105] are both known to regulate translation.

#### MAPK signaling as a translation initiation regulator

Other Ser/Thr kinases that regulate many essential processes are the MAPKs, which signal through a module composed of conserved, sequentially acting kinases [106]. Both the RAS/ERK and the p38/MAPK pathways converge on RNA translation and are activated by a wide range of stimuli [107, 108]. The effectors of MAPK signaling, the MAPK‐activated protein kinases (MAPKAPKs), include the p90 ribosomal S6 kinases (RSKs) [109] and the MAPK‐interacting kinases (MNKs) [110]. Since the RSKs and the MNKs are directly implicated in the regulation of mRNA translation initiation, the dysregulation of the MAPK signaling via these effectors is suggested to be important during CRC tumorigenesis (Fig. 4) [111].

MNKs are MAPKAPKs that are activated in response to carcinogenic signals and cellular stress, respectively [112]. They mediate the phosphorylation of eIF4E through binding with eIF4G [113]. Unlike the overexpression of eIF4E, which directly stimulates tumor initiation, eIF4E phosphorylation facilitates tumor progression through the translation of a selective subset of mRNAs critical for extracellular remodeling, metastasis, and tumor inflammation [27, 114]. In CRC, the specific inhibition of MNK1/2, and thereby the inhibition of eIF4E phosphorylation, results in reduced tumor cell viability and tumor growth, suggesting the importance of the MNK/eIF4E axis in CRC tumorigenesis [115, 116].

Interestingly, it has recently been shown in mouse models of CRC that KRAS activation results in the rewiring of translation, via the MNK‐mediated phosphorylation of eIF4E. This results in the increased binding to, and translation of, c‐Myc, driving proliferation. The same study showed that this was a druggable weakness in these tumors, with the combination of MNK and mTOR inhibition significantly inhibiting proliferation and extending survival. That study also showed that roughly 45% of CRC patients had a high level of signaling in both of these pathways, and that this correlated with decreased survival, suggesting that this may be a viable therapeutic option [69].

Alongside the MNKs, other MAPKAPKs that are known to be important in the dysregulation of the translation initiation in CRC are the RSKs. Increasing evidence suggests that the hyperactivation of RSK1, RSK2, and RSK4 plays a significant role in the oncogenic reprogramming of the translation initiation machinery in CRC [117, 118, 119].

## Dysregulation of the translation initiation machinery in colorectal cancer

Even though the upstream regulation of the translation initiation machinery has been shown to play a major role during tumorigenesis in CRC, the aberrant function of the individual eIFs is also documented to cause translational reprogramming during tumor initiation and/or maintenance. While the exact mechanism of translational reprogramming by the eIFs remains to be fully elucidated, compelling evidence suggests that the dysregulation of these components is essential to almost all oncogenic cellular processes (reviewed in Ref. [11, 70]). Moreover, since most of the eIFs exhibit increased activity in tumorigenic cells, it has been suggested that cancer cells are ‘addicted’ to the elevated translational activity [120]. For example, it has been shown that the expression of eIFs 1, 5, and 6 are correlated with CRC progression and that they may have therapeutic potential [121]. Additionally, several studies have associated expression of various eIF3 subunits with CRC survival [55, 122, 123, 124]. In this review, however, we will focus on the most commonly described alterations found in the individual components of the translation machinery and their significant role during CRC tumorigenesis. In particular, these components include the function and assembly of the eIF4F complex, the importance of key component eIF4A, and the cellular response to psychopathological stressors through eIF2α and eIF2B.

### eIF4E and 4E‐BPs

Various oncogenic signaling pathways are able to enhance and alter the translation initiation machinery, predominantly through alterations in the eIF4F complex [10]. Among the most studied members of this complex are initiation factor eIF4E and its binding partner 4E‐BP, which both fulfill a crucial role during translational control in CRC. As previously described, the hyperactivation of eIF4E is by itself sufficient to drive oncogenic transformation and tumorigenesis in CRC [125]. Upstream signaling pathways can promote eIF4E hyperactivation in multiple ways [112, 114, 126, 127]. Interestingly, however, both the expression and the phosphorylation of 4E‐BP1 are increased in CRC [102, 128]. As phosphorylation is an inhibitory event, this is counterintuitive, and it is probable that the resultant modulation of translation initiation is context‐dependent.

Once activated, eIF4E overexpression enables oncogenic transformation via the translation of a select set of mRNA transcripts involved in cellular proliferation (*c‐Myc* [69, 129], *CCND1* [130]), cell growth (*ODC1* [131]) and in angiogenesis (*VEGFA* [132]). Alongside this, eIF4E hyperphosphorylation facilitates metastasis through epithelial‐to‐mesenchymal transition, tumor inflammation, and extracellular remodeling [133]. While hyperphosphorylation of eIF4E is shown to be a rather early event during CRC carcinogenesis [117], eIF4E overexpression is a progressive process throughout tumor development [134, 135]. Evidence suggests that the high abundance of the eIF4E protein is strongly related to the histological type of lesion, with colorectal adenocarcinomas showing the highest eIF4E expression [135]. Aside from this, the overexpression of this initiation factor is also suggested to be associated with a higher risk of metastasis [136, 137], chemoresistance [138], and poor clinical prognosis in CRC [139].

### eIF4A and PDCD4

In addition to the upregulation of eIF4E, the overexpression of RNA helicase eIF4A also alters protein synthesis and this has been shown to be of great importance in cancer development [140]. Initiation factor eIF4A is considered to be the enzymatic core of the eIF4F complex and facilitates the recruitment and scanning of the 43S PIC along the mRNA 5’UTR in an ATP‐dependent manner [141, 142]. Compelling evidence suggests that the overexpression of this factor promotes the translation of many oncogenic mRNAs involved in cell proliferation, cell survival, and angiogenesis [140]. These tend to have highly structured 5’UTRs [142] and show an enrichment of G‐quadruplex structures (reviewed in Ref. [143]). The most important negative regulator of eIF4A is its binding partner PDCD4, which further limits the expression levels of eIF4A, preventing it from participating in translation initiation.

In CRC, the activity of tumor suppressor PDCD4 is found to be downregulated through its phosphorylation by S6K1, which, as a result, enhances the eIF4A availability and activity [105, 143]. This increased expression of eIF4A promotes the translation of c‐Myc, Cyclin D1, and BCL‐2 [140, 144]. Accordingly, the specific inhibition of eIF4A reduces intestinal tumor growth and CRC cell viability [145], but additionally increases various antitumorigenic effects in CRC [146, 147], underlining its importance in CRC tumorigenesis.

### eIF2α and eIF2B

Another essential complex regulating translation initiation in CRC is the TC, which, in response to stressors, can promote the ISR via eIF2α phosphorylation [48]. Phosphorylation of eIF2α enhances its affinity for eIF2B, which thereby sequesters the eIF2B activity and availability required for TC formation and as a result limits translation initiation [50, 51, 52]. While it is normally only transiently activated, under oncogenic conditions its expression levels are often found to be constitutively elevated [148]. In CRC, and cancer in general, this phenomenon can be explained by an increase in various tumorigenic stimuli, such as oncogene activation, ER stress, and nutrient deprivation [149]. Counterintuitively, both the phosphorylated and nonphosphorylated forms of eIF2α are found to be upregulated in CRC; however, the functional relevance of this is not known [148, 150]. What is known is that the phosphorylation status of eIF2α determines its function global protein synthesis and the ISR, and elevated phosphorylation increases oncogenic potential in APC‐deficient models of CRC. This suggests that the key role of eIF2α is in the global inhibition of translation, but this has not been proven thus far [72, 150].

The phosphorylation status of eIF2α can be modulated via the activation of eIF2α kinases PERK, GCN2, PKR, and HRI, depending on the stimulus [48]. PKR has been shown to be increasingly expressed in CRC progression [151], but the most studied eIF2α kinases are PERK (classically activated by misfolded proteins in the ER [152]), and GCN2 (stimulated as a result of amino acid deprivation in the cell [153]). Whereas the endogenous expression of p‐eIF2α is known for its cytoprotective role and its function in cellular adaptation [149], in cancerous circumstances, the phosphorylation of eIF2α by PERK or GCN2 is thought to increase cancer cell survival, tumor growth, and angiogenesis, at least partially through the buffering of translation in order to prevent nutrient depletion [154]. In an aggressive prostate cancer mouse model, it has been shown that translation inhibition by eIF2α phosphorylation buffers the cancer cells from dramatic unrestrained increases in protein production, allowing continued tumor growth [155]. It has also been shown that such stress‐related adaptive translational responses drive plasticity in breast cancer models, through translational reprogramming and the specific regulation of stem cell‐related mRNA isoforms with alternative 5’UTRs [156].

In CRC, the transmembrane receptor PERK can phosphorylate eIF2α which can result in both the temporal inhibition of translation initiation and the translation of a small subset of mRNAs involved in response to ER stress [157], such as *ATF4* [158]. The constitutive phosphorylation of eIF2α by PERK is known to result in the loss of intestinal stem cell differentiation and stemness [159], but might also play an important role in chemoresistance [160]. The BCL‐2 inhibitor SPARC has also been shown to play a role in CRC response to ER stress, through its interaction with GRP78, a master regulator of the response to ER stress. Downregulation of SPARC appears to allow CRC cells avoid apoptosis, underlining the highly regulated nature of this response [161].

GCN2 is activated in response to amino acid deprivation and is crucial for binding uncharged tRNAs [153]. The enhanced phosphorylation of eIF2α by GCN2, in combination with elevated c‐Myc and cellular stress, is an important oncogenic mechanism in APC‐deficient cells [72]. Importantly, eIF2α phosphorylation via both PERK and GCN2 enhances the expression of selected proteins, in particular that of ATF4, which enables tumor cell survival and adaptation in c‐Myc‐driven cancer types, including CRC [92, 162]. Although the function of eIF2α and eIF2B within translational control is found to be relatively clear during conditions of cell stress, their roles in tumorigenesis are still being understood. As described above, we are beginning to reveal the function of eIF2α‐mediated translational repression in cancer, but further studies are still needed.

## Conclusion

Growing evidence demonstrates the importance of the regulatory mechanisms controlling RNA translation in cancer. While both initiation and elongation have been shown to be altered in cancer, its rate‐limiting phase (translation initiation) is of particular importance. Over the last few decades, many initiation factors, as well as the signaling pathways converting into the translation machinery, have been found to contribute to the development and progression of numerous human cancer types, showing the significance of strict translational control. Although the exact underlying mechanisms by which certain translation initiation factors are involved in tumorigenesis remain to be elucidated, strong progress has been made and has revealed new insights into translational reprogramming and its function as a strong oncogenic driver, especially in CRC. The elongation phase of translation has also been shown to play a vital role in intestinal cancer in mice. This is thought to be primarily involved in the initial stages of tumorigenesis, and its role in latter stages is not yet known. However, it emphasizes that the dysregulation of whole translational regulatory circuitry in CRC may serve as the foundation for pathogenesis and etiology of the disease.

Altogether, an outstanding amount of data has indicated the critical role of the translation initiation machinery and its regulatory circuitry in cancer initiation and development, with the majority showing not only its impact on cell survival, growth, and proliferation but also the cellular response to stress and angiogenesis. However, to date, no approved treatments targeting the translation machinery are utilized for therapeutic use in CRC patients, yet even though a few promising targeting strategies are advanced to clinical trials. This can partially be explained by the fact that the translation fulfills an essential and general housekeeping function in the human body, which, as consequence, could interfere with these therapeutic treatments leading to systemic toxicity. Additionally, significant knowledge, regarding the exact underlying molecular and cellular mechanisms involved in this translational reprogramming, needs to be elucidated in order to develop more successful therapeutic treatments. Accordingly, there is a need for therapeutic approaches selectively and specifically targeting proteins involved in the translational reprogramming of tumorigenic cells in the intestine. Ultimately, as research continues to obtain a more discrete understanding of the regulatory mechanisms underlying translation initiation of the cancer genome, this will open the opportunity to develop and provide novel therapeutic approaches for CRC patients.

## Conflict of interest

The authors declare no conflict of interest.

## Author contributions

EM and WJF conceived, wrote, and revised the manuscript.

### PEER REVIEW

The peer review history for this article is available at https://publons.com/publon/10.1111/febs.15690.

[Correction added on 24 March 2021, after first online publication: URL for peer review history has been corrected.]
